# Multispectral imaging and unmanned aerial systems for cotton plant phenotyping

**DOI:** 10.1371/journal.pone.0205083

**Published:** 2019-02-27

**Authors:** Rui Xu, Changying Li, Andrew H. Paterson

**Affiliations:** 1 Bio-Sensing and Instrumentation Laboratory, College of Engineering, University of Georgia, Athens, Georgia, United States of America; 2 Plant Genome Mapping Laboratory, University of Georgia, Athens, Georgia, United States of America; Texas A&M University, UNITED STATES

## Abstract

This paper demonstrates the application of aerial multispectral images in cotton plant phenotyping. Four phenotypic traits (plant height, canopy cover, vegetation index, and flower) were measured from multispectral images captured by a multispectral camera on an unmanned aerial system. Data were collected on eight different days from two fields. Ortho-mosaic and digital elevation models (DEM) were constructed from the raw images using the structure from motion (SfM) algorithm. A data processing pipeline was developed to calculate plant height using the ortho-mosaic and DEM. Six ground calibration targets (GCTs) were used to correct the error of the calculated plant height caused by the georeferencing error of the DEM. Plant heights were measured manually to validate the heights predicted from the imaging method. The error in estimation of the maximum height of each plot ranged from -40.4 to 13.5 cm among six datasets, all of which showed strong linear relationships with the manual measurement (*R*^2^ > 0.89). Plot canopy was separated from the soil based on the DEM and normalized differential vegetation index (NDVI). Canopy cover and mean canopy NDVI were calculated to show canopy growth over time and the correlation between the two indices was investigated. The spectral responses of the ground, leaves, cotton flower, and ground shade were analyzed and detection of cotton flowers was satisfactory using a support vector machine (SVM). This study demonstrated the potential of using aerial multispectral images for high throughput phenotyping of important cotton phenotypic traits in the field.

## Introduction

To meet the demands of the predicted global population of 9 billion by the year 2050, current crop production must double by that time [1]. This is a tall order, challenging plant breeders to find genotypes with high yield, as well as high-stress tolerance to adapt to the changing climate in the next 30 years. Recent technological advances in molecular biology have offered tools that can significantly accelerate the breeding process [2]. However, phenotyping has become the bottleneck to using these new technologies to their full potential. Screening genotypes—in order to select those with the most desirable traits—heavily relies on the ability to characterize and measure traits [3]. Therefore, many plant breeders and engineers recognize the need for a high-throughput phenotyping (HTP) system capable of efficiently and accurately measuring phenotypic traits [3, 4].

The development of an HTP system is challenging in both the platform design and the associated data processing methods. Development of a field-based high-throughput phenotyping system (FHTPS) is even more challenging due to heterogeneous field conditions and uncontrolled environments, which can affect the data quality and make results difficult to interpret [4]. Some FHTPSs utilize ground vehicles (either tractors or robotic platforms) equipped with sensors to acquire data [5–7]. Ground vehicles have the advantage of carrying large payloads and can easily include multiple sensors at a time. In addition, ground platforms can control the data collection environment (such as light conditions) to some degree with well-designed enclosures, thus guaranteeing data quality. However, ground platforms also have several disadvantages: the data collection speed is low, frequent data collection can cause soil compaction, the wheels can damage the crop, and the platform is difficult to adjust for a wide range of crops once the design is fixed. As an alternative to ground platforms, unmanned aerial systems (UAS) can address the disadvantages of ground platforms to some degree. Compared to ground platforms, UAS can provide superior data acquisition speed and larger spatial coverage [8]. Since no interaction exists between the plots and the UAS, UAS can be easily adapted to different types of crops and different growth stages [9]. Furthermore, UAS can be automatically controlled by its onboard autopilot system, and therefore requires less human intervention during data collection.

Traditional precision agriculture and remote sensing studies have shown the broad applications of satellite or airborne images for crop management, stress detection, and yield estimation [10]. The general research methodology and data analysis techniques from remote sensing can be readily used for aerial images with high spatial and temporal resolutions taken by UAS, which can significantly benefit high-throughput phenotyping research. Simple traits such as plant height and canopy cover can be measured using aerial imaging to monitor crop growth and estimate the final yield. Plant height measured from crop surface model generated by aerial color images was used to develop regression models to predict the biomass for barley and the best model achieved a relative error of 54.04% [11]. The regression model was improved by combining vegetation indices from ground-based hyperspectral reflectance data, achieving 44.43% relative error on biomass prediction [12]. Spectral response of the canopy—measured using aerial hyperspectral or multispectral imaging—can be used to detect biotic and abiotic stress of the plant [13]. It was shown that Huanglongbing-infected citrus trees can be detected using various vegetation indices with 85% classification accuracy [14]. Tomato spot wilt disease in peanuts was best detected by normalized difference red edge (NDRE) using multispectral imaging [15]. Previous studies showed that aerial hyperspectral or multispectral imaging was useful to detect water stress [16, 17]. Normalized difference vegetation index (NDVI) showed highest correlation (*R*^2^ = 0.68) with water potential in grape vineyard among seventeen vegetation indices, which showed NDVI could be a good indicator for long-term water deficits [18]. NDVI can also be used to separate olive tree crown from the soil in aerial images [19]. Canopy temperature is an indicator of water stress, but it is extremely sensitive to small changes in the environment, making the ability of the UAS to quickly and repeatedly measure many plots preferable to ground platforms for measuring this trait [20, 21]. Traits that require continuous measurements, such as flowering time, are also well suited to aerial imaging.

Although applications of UAS in agriculture have been studied for a wide range of crops, only a few applications of aerial imaging for cotton have been reported despite the importance of cotton as an industrial crop for producing natural fibers. One study used low-altitude aerial color images to estimate plant height and yield and they found that the cotton unit coverage can be better correlated with yield than plant height [22]. Another study used aerial color images to monitor cotton growth by measuring plant height and canopy cover and estimate the yield from them using various regression models [23]. The result showed good correlation between plant height and canopy cover, but a low correlation between the estimated yield and observed yield (*R*^2^ = 0.5 for the best model). Due to the lack of manual measurement, the accuracy of the plant height and canopy cover was unknown in this study. Despite the two relevant studies, the use of aerial images to measure other important phenotypic traits such as flowering and boll opening has received little attention. There is still a gap in knowledge and technical development that hinders capitalizing the latest UAS and imaging technologies for cotton breeding.

To explore the potential of cotton phenotyping using UAS, this paper focuses on measuring multiple traits including plant height, canopy cover, vegetation index, and flower detection using a multispectral camera. Specifically, this paper develops a method to measure cotton plant heights with a UAS-based FHTP system, validating the accuracy of the method with manual measurements. Canopy cover, the proportion of the ground covered by the crop canopy, will be derived based on the crop surface model and the normalized differential vegetation index (NDVI). The feasibility of flower detection based on the spectral response will be explored, providing a foundation for future quantification of the distribution of flowering over the growing season.

## Materials and methods

### Test fields

**Fig 1:**
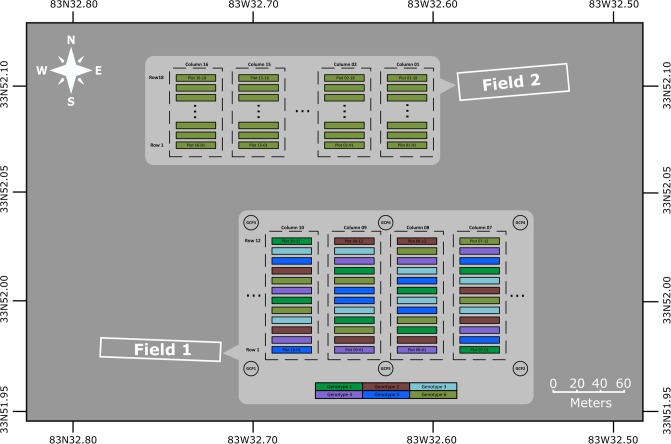
Location and plot layout of the two test fields. The genotypes of the plots in field 1 were indicated in different colors.

The study was carried out on Plant Science Farm at University Georgia, Athens campus and the owner of the land gave permission to conduct the study on this site. Furthermore, the field studies did not involve endangered or protected species. Two test fields were used in this study, both of which located at the University of Georgia Plant Sciences Farm (33°52′02.8”*N*, 83°32′39.5”*W*) in Watkinsville, GA (Fig 1). A total of 240 plots of cotton were planted in field 1 on June 15, 2015, arranged in 20 columns and 12 rows, with a 1.8 m alley between each column. Each plot was 3 m long and 0.9 m wide, and 15 seeds were planted in each plot. Six genotypes were used in field 1. Genotype 1 to 5 were provided by a cotton breeder from the University of Georgia, Tifton Campus. They were GA2011158, GA230, GA2010074, GA2009037 and GA2009100, respectively. Genotype 6 was Americot UA48. Each column had twelve plots and each genotype with two replicates were randomly assigned to each column. The germination rate of the plots varied from 13% to 100%, resulting in variance in the plot density.

Due to lack of high accurate surveying tool, no ground control point was used to correct the aerial triangulation error of the crop surface model. Instead, six round stainless steel plates with a diameter of 30 cm, which were referred as ground calibration targets (GCTs), were raised around 1.5 m above the ground using plastic pipes and placed around the border of the test field. The GCTs were painted in black patterns on white background (S1 Fig). Those GCTs were used to evaluate and calibrate the crop surface model. To avoid the power lines over the field during data collection, only 48 plots (from column 7 to column 10) were selected as test plots. However, the plants from row 1 to row 6 were seriously damaged by a tractor after October 7. Therefore, only 24 plots from row 7 to row 12 were used for data analysis since October 7.

Because of late planting, plants in field 1 did not produce many flowers over the season. Therefore, we used field 2 to collect cotton flower images. Field 2 had 288 plots arranged in 16 columns and 18 rows and it was based on complete random block design. We selected 22 plots to develop and validate the method for cotton flower detection.

### Image collection

Aerial multispectral images of the two fields were acquired using an octocopter (s1000+, DJI, China) carrying a lightweight multispectral camera (RedEdge, MicaSense, WA). The multispectral camera was mounted on a gimbal and faced the ground. The multispectral camera has five global shutter camera modules that provide five band images—blue, green, red, near-infrared (NIR), and red edge (RE). The center wavelengths of each band are 475 nm, 560 nm, 668 nm, 840 nm and 717 nm, respectively. The multispectral camera has a low accuracy GPS (2.5 m) which can record the geographic coordinates of the image. The size of the band image is 1280×800. In order to validate flower detection results from multispectral images, aerial color images of field 2 were acquired (at the same time as the multispectral images) using a color camera (Lumix DMC-G6KK, Panasonic, Japan) that provides a maximum image size of 4624×3472.

To collect the aerial multispectral imaging data, the drone flew autonomously along a preset flight path at a speed of 2.5 m/s and a height of 20 m above ground level (AGL) for field 1, achieving ∼1.5 cm ground sample distance for multispectral images. The endlap and sidelap of the collected multispectral images were over 85%. To image cotton flowers as closely as possible, while maintaining the same flight speed, we set the flight height at 15 m AGL for field 2, which was the minimum height that downdraft of the UAV did not disturb the plants and images had enough overlap. In reality, the drone flew lower than 15 m due to the wind condition and error of altitude sensor, which gave the actual ground sample distance of ∼0.8 cm for the multispectral images. The endlap was over 80% and sidelap was over 50% for the multispectral images. The color and multispectral cameras were triggered simultaneously at the frequency of 1 Hz with a customized triggering circuit. The exposure time and aperture were manually set for the color camera according to the light conditions of the field to get the best image quality and other parameters used auto-settings. At each data collection, a reflectance panel provided by MicaSense was imaged before and after the flight. In total, we collected one set of images for the field 2 and seven sets of images for the field 1 (Table 1). The field 2 images were used to explore the feasibility of flower detection using multispectral images, while the field 1 images were used to calculate plant heights, canopy cover, and vegetation index.

**Table 1:** Data collection summary.

Field	Date	Time	Flight height	Flight speed	Flight time
Field 2	8/28/2015	12:00 PM	15 m	2.5 m/s	6 min
Field 1	9/18/2015	1:00 PM	20 m	2.5 m/s	5 min
	9/30/2015	12:00 PM	20 m	2.5 m/s	6 min
	10/7/2015	4:00 PM	20 m	2.5 m/s	5 min
	10/16/2015	11:00 AM	20 m	2.5 m/s	5 min
	10/19/2015	2:00 PM	20 m	2.5 m/s	5 min
	10/23/2015	10:00 AM	20 m	2.5 m/s	5 min
	10/30/2015	10:00 AM	20 m	2.5 m/s	5 min

### Reference measurement

On each data collection day (except September 18, 2015), the height of each plot in the field 1 was manually measured as the ground truth. For each plot, individual plants were measured using a ruler and the height of the tallest plant within the plot was used as the maximum plot height. All 48 plots were measured on September 30 and October 7, and 24 plots (from rows 7 through 12) were measured on the other four days. The vertical distance between the edge of the GCT to the ground was measured four times using a ruler with millimeter accuracy at even spacing along the edge of the GCT and the average value of the four measurements was used as the GCT height reference. The manual measurements of plant height were used to calculate the accuracy of results from aerial images.

### Plot height, canopy cover and vegetation index extraction

**Fig 2:**
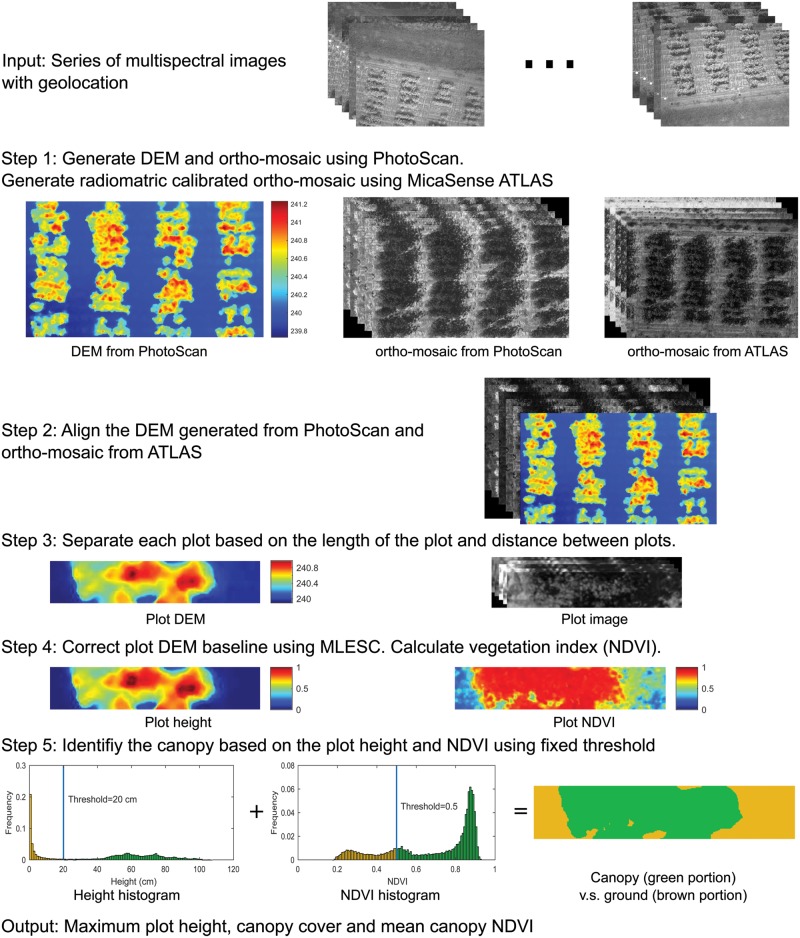
Data processing flowchart. DEM: digital elevation model. MLESC: maximum likelihood estimation sample consensus. NDVI: normalized difference vegetation index.

Multispectral images from the field 1 were used to calculate plot height, canopy cover, and vegetation index. The data processing flowchart consisted of five steps (Fig 2). The first step was to perform aerial triangulation and generate the digital elevation model (DEM) and ortho-mosaic. Two software/services were used in this step: PhotoScan and MicaSense ATLAS. Both of them can generate DEM and ortho-mosaic. The PhotoScan (PhotoScan Professional 1.2.6, Agisoft LLC, Russia) can generate better DEM with higher resolution than MicaSense ATLAS (atlas.micasense.com). However, the ortho-mosaic from PhotoScan is not radiometrically calibrated, resulting in incorrect vegetation indices. In contrast, the ortho-mosaic from ATLAS is radiometrically calibrated using the images of the reflectance panel. Therefore, we used the DEM generated from PhotoScan, and the ortho-mosaic from ATLAS for the subsequent data processing. The processing time for each step in PhotoScan was summarized in Table 2.

**Table 2:** Summary of the image processing time for generating ortho-mosaic and DEM in PhotoScan. Dense cloud and DEM was not built for 8/28 dataset since the ortho-mosaic was used for flower detection.

Dataset	Images	Align photos	Build dense cloud	Build ortho-mosaic	Build DEM	Total time
8/28	230	55 min, 14 sec	-	1 min, 46 sec	-	57 min
9/18	178	39 min, 55 sec	24 min, 57 sec	1 min, 46 sec	6 sec	66 min, 44 sec
9/30	171	41 min, 26 sec	31 min, 10 sec	1 min, 17 sec	6 sec	73 min, 59 sec
10/07	134	30 min, 8 sec	13 min, 31 sec	40 sec	6 sec	44 min, 25 s
10/16	137	31 min, 36 sec	19 min, 24 sec	49 sec	5 sec	51 min, 54 sec
10/19	135	29 min, 28 sec	15 min, 27 sec	52 sec	6 sec	45 min, 53 sec
10/23	114	22 min, 34 sec	9 min, 40 sec	2 min, 15 sec	6 sec	32 min, 35 sec
10/30	132	28 min, 13 sec	12 min, 35 sec	2 min, 20 sec	6 sec	43 min, 14 sec

The second step was to align the PhotoScan DEM and ATLAS mosaic using OpenCV (OpenCV 2.5). The scale-invariant feature transform (SIFT) image features were first calculated for each band image of the PhotoScan and ATLAS ortho-mosaics and their common image features were matched. Then the projective transformation from ATLAS ortho-mosaic to PhotoScan ortho-mosaic was calculated based on the locations of the common feature points. Finally, the ATLAS ortho-mosaic was transformed to the image frame of the PhotoScan ortho-mosaic, which is same as the PhotoScan DEM, using the projective transformation. Then the ATLAS ortho-mosaic and the DEM were aligned pixel by pixel.

The third step was plot segmentation, which was to divide the entire DEM and ortho-mosaic into individual plots (plot DEM and plot image) based on plot length and width. As a result, each plot-level DEM or ortho-mosaic has only plants and the surrounding soil. The area of the plot DEM and plot images were kept the same. The pixel value of the DEM indicates the altitude above sea level, therefore, the fourth step was to adjust plot DEMs so that the pixel value represented the relative height from the ground. For this purpose, the maximum likelihood estimation sample consensus (MLESC) algorithm was used on the DEM to find the best fitting plane to represent the ground surface since the ground within one plot can be assumed to be flat [24]. The MLESC was applied to the ground pixels whose normalized intensity on the red band image was larger than 0.4. The threshold was chosen arbitrarily but the misclassified ground pixels due to the threshold will not affected the ground surface significantly because the MLESC is robust to outliers. The threshold of MLESC was set to 0.1 m. The ground surface was subtracted from the DEM to get the relative height. Meanwhile, the vegetation index for each plot was calculated. Several vegetation indices can be derived from the five bands of the multispectral images, such as normalized difference vegetation index (NDVI) and normalized difference red edge (NDRE). A complete list of vegetation indices can be found in Torino et al.’s study [25]. In our study, only the NDVI was calculated using the red (R) and near-infrared band (NIR) using Eq 1.

$$\begin{array}{c} NDVI=\frac{NIR_{840}-R_{668}}{NIR_{840}+R_{668}} \end{array}$$ (1)

The subscript indicates the wavelength (nm).

The fifth step was to separate the canopy from the ground using plot height and plot NDVI by a threshold method. A threshold of 0.5 for NDVI was used to separate the vegetation from the soil [26] and a threshold of 20 cm for DEM was used to remove weeds. The maximum plot height was calculated as the largest height value within the canopy. When calculating the maximum plot height, only the central 75% of the canopy was used to avoid pixels from nearby plots. The canopy cover was calculated as the ratio of the number of canopy pixels to the total pixels of the plot. The NDVI of the plot was calculated using the mean NDVI value of the canopy.

### GCT height

For each dataset, the height of each GCT was calculated from the DEM following the same procedure as the plot height in order to evaluate the accuracy of the model and height calculation algorithm. However, due to the shape of the GCT, the last step was replaced by calculating the average GCT pixel values as the height of the GCT.

### Flower detection

Flower detection was performed using the field 2 image set. The ortho-mosaic was first generated using PhotoScan and was divided into plot images using the same procedure as in the height calculation. For each plot image, each pixel was classified into four different categories (flower, canopy, ground, ground shade) based on the raw pixel value using support vector machine type 1 (C-SVM) [27]. The SVM can return a categorical label and probability of being in each category. Using the criteria of the probability being in flower category larger than 0.75 rather than the categorical label to classify a pixel as a flower pixel was found helpful to prevent misclassifying some leave spots with high reflectance as flower pixels. The flower pixels generated a flower mask for each plot. The number of flowers was obtained by counting the connected components in the flower mask. The SVM was trained using the raw digital count of the pixels manually selected from the images for each category. The penalty of the C-SVM was set to 1, and the kernel function was Gaussian radial basis function with a gamma value of 0.2. The classification accuracy of the SVM was examined using four-fold cross validation. The number of flowers detected from the multispectral image was compared with the results of manual identification using the corresponding color image.

### Statistical analysis

The error of the maximum plot height was calculated by the difference between the calculated heights (CH) and the measured height (MH). Root mean square error (RMSE) and relative root mean squared error (R-RMSE) was calculated for each dataset using Eqs 2 and 3. Linear regression between the measured height and calculated height was performed and the coefficient of determination (*R*^2^) was calculated for each dataset. All the statistical analysis were done in MATLAB (2016a, MathWorks).

$$\begin{array}{c} RMSE=\sqrt{\frac{\sum_{i=1}^{N}{\left(CH_{i}-MH_{i}\right)}^{2}}{N}} \end{array}$$ (2)

$$\begin{array}{c} R\text{-}RMSE=\sqrt{\frac{\sum_{i=1}^{N}{\left(\frac{CH_{i}}{MH_{i}}-1\right)}^{2}}{N}} \end{array}$$ (3)

Where N is the total number of plots.

## Results

### GCT height

**Fig 3:**
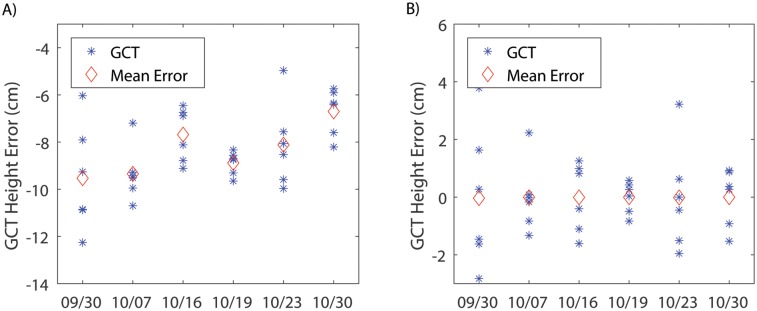
Accuracy of height measurements of the ground calibration targets by the imaging method. **A**) The error in calculating ground calibration target heights before error correction. **B**) The error in calculating ground calibration target heights after error correction.

The accuracy of calculated plot heights was affected by the accuracy of the DEM. The heights of GCTs were used to examine the accuracy of the DEM because the rigid body of the GCTs was less prone to error than plants. The error of the GCT height varied for different date and among the GCTs (Fig 3A). The mean errors for 9/30, 10/7, 10/16, 10/19, 10/23 and 10/30 were -9.8 cm, -9.4 cm, -7.6 cm, -8.7 cm, -8.3 cm and -6.7 cm, respectively. This suggested that there were systematic errors in the DEM that vary by day. The systematic errors resulted from the georeferencing error of the images and triangulation error during dense point cloud generation. The common practice for error correction is to use accurately surveyed ground control points to correct the error introduced by inaccurate georeferencing of the aerial images, or to scale the DEM using a reference object with its true size. The second method was adopted to correct the systematic error by calculating the scale factor between the calculated GCT height and the measured height. After error correction, the errors of the GCT heights were reduced to -2.6–3.2 cm (Fig 3B).

### Maximum plot height

**Fig 4:**
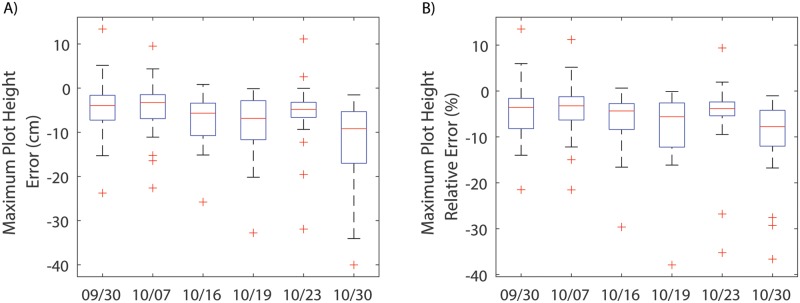
Error of the calculated maximum plot height. **A**) The absolute error in calculating maximum plot heights. **B**) The relative error in calculating maximum plot height.

**Fig 5:**
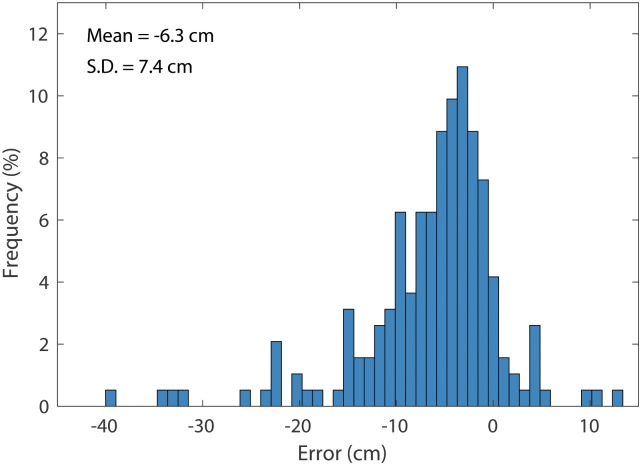
The error distribution for the maximum plot height.

**Fig 6:**
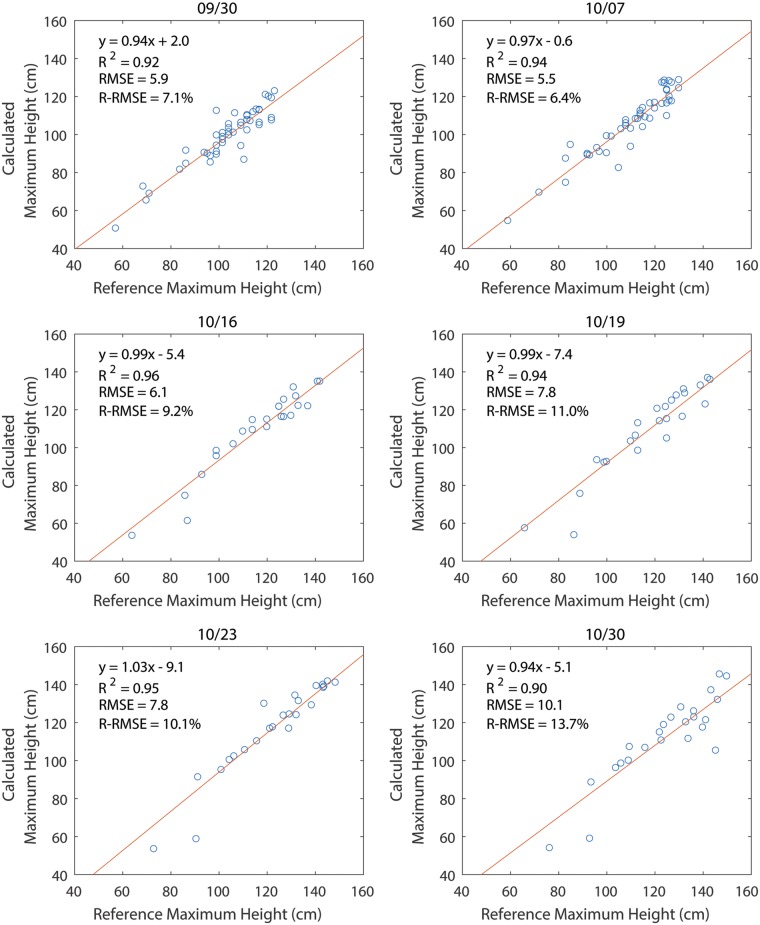
Correlation between calculated maximum plot heights and manually measured maximum plot heights using a linear model.

The calculated plant heights were adjusted using the scale factor from the GCTs. After correction, the mean errors of the maximum height for 9/30, 10/07, 10/16, 10/19, 10/23, and 10/30 were -4.2 cm, -4.3 cm, -7.1 cm, -8.0 cm, -5.6 cm, and -12.1 cm, respectively (Fig 4A). The mean of the relative error for each day ranged from -3.9% to -10.2% (Fig 4B). This suggested that the calculated maximum plot heights were consistently smaller than the reference height measured manually, which is consistent with the findings in Huang et al.’s study [22]. The errors for all data sets, ranging from -39.0 to 13.5 cm, approximately followed a normal distribution with a mean of -6.2 cm and a standard deviation of 7.5 cm (Fig 5). The coefficient of determination (*R*^2^) between the calculated maximum plot heights and manually measured maximum plot heights was between 0.9 and 0.96, showing a strong linear relationship between calculated heights and the reference heights (Fig 6).

### Canopy cover and vegetation index

Canopy cover indicates the proportion of the ground area covered by the canopy, which can be used to quantify canopy development over time (Fig A in S2 Fig). Ideally over time the canopy cover increased until reaching full coverage of the ground. Plots with larger canopy cover can capture more sunlight, which potentially can produce more cotton fiber. The NDVI usually increases as the canopy develops, reaching a maximum the canopy fully develops, then declines as the plant remobilizes resources into the bolls and starts to defoliate (Fig B in S2 Fig).

**Fig 7:**
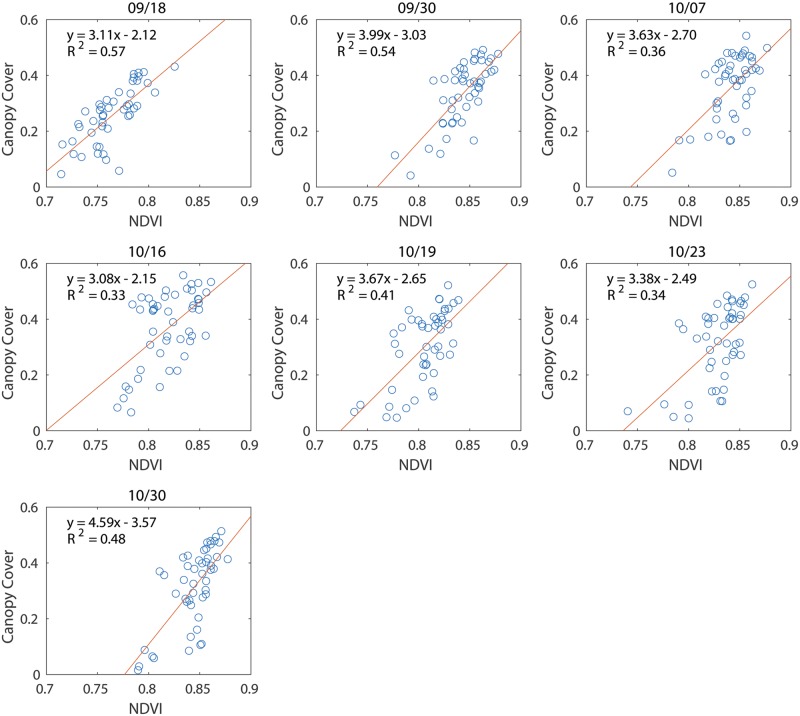
Correlation between NDVI and canopy cover on different dates.

Studies have shown that NDVI has a high linear correlation (*R*^2^ > 0.6) with leaf area index (LAI), which is an important index to quantify plant canopy [28]. Since NDVI and LAI are both correlated with canopy cover, a linear relationship was found between NDVI and canopy cover (Fig 7). The coefficient of determination(*R*^2^) was low because the data collection was taken in the late vegetative stage, where correlation between LAI and NDVI was declined. Another reason of the low *R*^2^ could be due to the late planting that caused the cotton plants to grow differently from normal growth. The linear relationship became weak after the canopy fully closed due to the defoliation of the leaves.

### Flower detection

**Fig 8:**
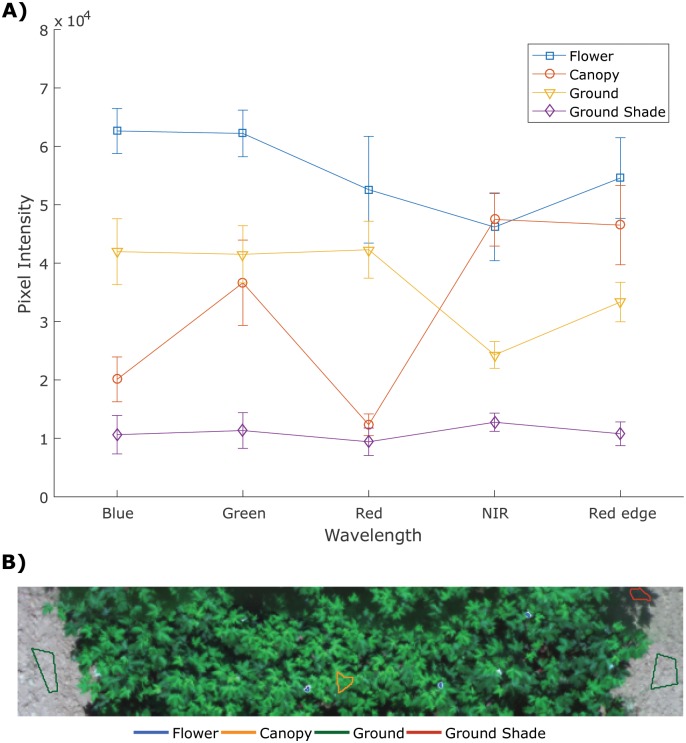
**A**) Pixel values of four objects (flower, canopy, ground, and ground shade). **B**) The four regions of interest in the image.

**Fig 9:**
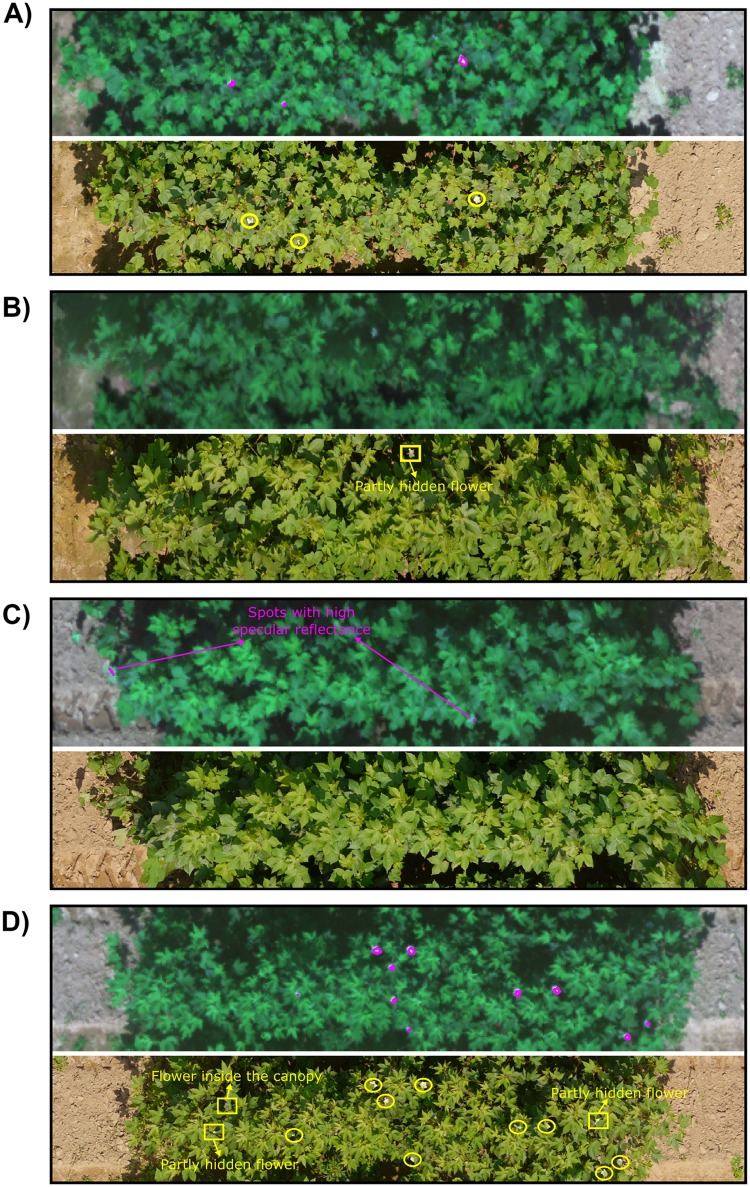
Example plots of the automatically detected flowers in the multispectral images (composited from the blue, red, and green band) and manually identified flowers in the color images. In the multispectral images (top image in each panel), the magenta lines indicate detected flowers. In the color images (bottom image in each panel), the yellow circles indicate detected flowers and the yellow squares indicate undetected flowers in multispectral images. **A**) All the flowers in the color image were detected in the multispectral image. **B**) The flower inside the canopy was not detected in the multispectral image. **C**) Spots with high specular reflectance from the leaves were misclassified as flowers in multispectral images while the color images showed no flowers. **D**) Most of the flowers in the color image were detected in the multispectral image. Two partly hidden flowers and one flower inside the canopy were not detected in the multispectral image.

Multispectral images showed the clear separation between flowers and other objects (canopy, ground and ground shade, Fig 8A). Since the multispectral images were taken under auto exposure for each channel, each band image always gave the best contrast between the plant and ground. Flowers showed the highest pixel value on the blue, green, and red channels; therefore, flowers can be clearly separated from other objects using these three channels. The ground was clearly separated from other objects on the NIR channel. Because of the clear separation of the spectrum between different categories, we were able to train the SVM with 100% classification accuracy for flower and non-flower classes for the training set at the pixel level. The detection results from multispectral images showed few false negatives and false positives (Table 3). In a few cases, all the flowers were detected and match with the color images (Fig 9A), while in most case, the flowers cannot be detected with 100% accuracy for several reasons. First, the shadow of the leaves decreased the pixel value of flowers inside the canopy, and the SVM could not detect those flowers because their spectrum was closer to the canopy spectrum, which caused false negatives (Fig 9B). Second, leaves with high specular reflectance could be misclassified as flowers because they gave high pixel values, and thus caused false positives (Fig 9C). Third, because of the resolution limitation of the multispectral images, a cotton flower was only several pixels large in the images. This increased the risk of missing small flowers or flowers partly hidden by leaves. In Fig 9D, two partly hidden flowers were not detected in the multispectral image.

**Table 3:** Comparison of the flower detection using multispectral images with the manual detection using color images.

Plot	Flowers in color image	Flowers detected by multispectral image		
		False negative (rate in percentage)	False positive (rate in percentage)	True positive (rate in percentage)
1	4	1(25)	0(0)	3(75)
2	1	0(0)	0(0)	1(100)
3	4	3(75)	0(0)	1(25)
4	3	1(33)	0(0)	2(67)
5	4	1(25)	0(0)	3(75)
6	4	1(25)	1(25)	2(50)
7	4	1(20)	1(20)	3(60)
8	7	2(22)	2(22)	5(56)
9	5	1(17)	1(17)	4(67)
10	4	1(20)	1(20)	3(60)
11	5	3(60)	0(0)	2(40)
12	4	2(50)	0(0)	2(50)
13	4	0(0)	3(43)	4(57)
14	9	0(0)	3(27)	8(73)
15	4	2(33)	0(0)	4(67)
16	3	2(67)	0(0)	1(33)
17	1	0(0)	1(50)	1(50)
18	1	1(100)	0(0)	0(0)
19	5	1(20)	0(0)	4(80)
20	5	1(17)	0(0)	5(83)
21	5	0(0)	1(17)	5(83)

## Discussion

This study demonstrated the success of using aerial multispectral images to measure plant height, canopy cover, and vegetation index, as well as the feasibility to detect cotton flowers. The UAS greatly improved the data collection throughput in the field and provided ability to continuously monitor cotton growth over the season.

In terms of accuracies for plant height measurement, the root mean squared error (RMSE) ranged from 5.7 cm to 10.4 cm (the relative root mean squared error (R-RMSE) ranged from 6.6% to 13.6%). Because of the lower ground sample distance, the accuracy is better than most of the studies on tree height measurement. One study achieved the RMSE between 20 and 45 cm (R-RMSE ranged from 6.55% to 19.24%) for olive tree height [19]. Another study measured single olive tree using multispectral imaging and the average error was 22 cm (6.32%) and 53 cm (15.55%) for 50 m and 100 m flight height, respectively [29]. Compared with similar studies on crops with lower height such as barley and wheat, the proposed method achieved a similar accuracy [11, 30]. The error of the height measurement is mostly contributed by the error of the DEM, which can be largely corrected using the GCTs. However, certain errors involved in the DEM generation process cannot be corrected in the case of maximum height. For example, the highest point may be smoothed out due to the size of the ground pixel or the depth filter during the construction of DEM because of the movement of plant leaves, resulting in generally lower value than the true maximum height. Another significant error source is the algorithm to extract height from DEM, where the plot segmentation and baseline correction introduced errors into the height calculation. During the plot segmentation, only the central 75% of the plot canopy was used to avoid the overlap with nearby plots, potentially excluding the highest points and resulting in a lower calculated maximum height than the manual measurement. The resulted errors can be as large as -40.4 cm, but most of the errors were within -20 cm to 20 cm. The baseline correction can correct the unevenness of the ground (soil contours left by the tractor), which could be an issue for manual measurement since the base of the plant changed with the soil contour. This can be revealed by the standard deviation of the difference between the ground plane and the DEM for the ground pixels which was in the range of 1–12 cm for plot DEMs from all data sets. Similar results were also found by Chu et al. that the bare soil areas have ~5 cm uncertainty in DEM [23].

**Fig 10:**
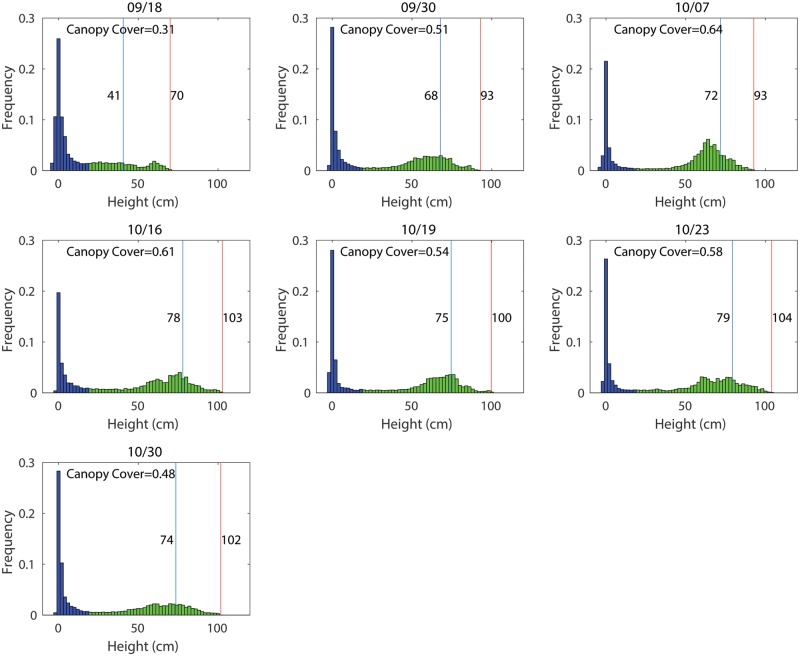
Histogram of the height for one plot over 42 days. Blue and green areas indicate the ground and canopy, respectively. Blue and red lines respectively indicate the 85th and 100th percentile (maximum) of canopy heights.

Because the maximum plot height can be affected by the above error sources, it may not accurately reflect the overall canopy for the plot. The DEM not only provides height information but also the spatial distribution of the height. In this case, the height histogram presents the distribution of the height, which can better describe the development of the canopy (Fig 10). For example, changes over time of the height from low to high indicate the growth of the canopy. The histogram can be divided into two parts using a threshold of 20 cm, where the left part and right part indicate the height distribution of the ground and canopy, respectively. The metrics for characterizing the histogram, such as mean, standard deviation, skewness, kurtosis, and percentile, can be used to characterize canopy height. These metrics can be directly obtained with existing statistical tools. For example, the 85th and 95th percentile of canopy height distribution within a plot were used to represent the height of corn and wheat [30, 31].

Flowering time is an important factor that effects the cotton yield [32]. Currently there is no high-throughput method to measure flowering time other than human visual evaluation in the field. The preliminary results have shown that the multispectral images have the potential to detect cotton flowers, which can be very helpful to continuously monitor the flower development over the season, although more research needs to be done to overcome its limitations. For example, the flowering count can be underestimated comparing to human evaluation because flowers hidden inside the canopy may not be captured by the image. Using oblique imagery to provide different views of the canopy to increase the chance to capture the flowers inside canopy could be a solution to solve the underestimation.

The main contribution of this study is that we developed a methodology to measure multiple phenotypic traits using multispectral images on a UAS. These image-derived traits were validated against manual measurements, providing confidence of our method (S1 Table). Compared to other methods used to calculate crop/tree heights, our method has two key improvements. First, it does not require the bare soil model that was used in other papers [11, 23]. It is particular useful for perennial crops and trees whose bare soil model cannot be measured. Second, using the fitted ground surface as baseline can compensate the unevenness of the ground terrain in comparison to the average or minimum height of the surrounding soil that were used in the tree height estimation studies [19, 29, 33]. Another unique contribution of this study is that we demonstrated the feasibility of detecting cotton flowers for the first time, making it possible to quantitatively monitor cotton flower development over time. We acknowledge that our methodology has certain limitations and some aspects can be improved. For example, the georeferencing error of the ortho-mosaic and DEM limited the usage of the automatic plot segmentation based on the geolocation of the plots. The error would be greatly reduced if accurately surveyed ground control points were used. The resolution of the multispectral camera used in this study was relatively low and it limited the accuracy of the result. In addition, two independent software/services were used to generate ortho-mosaics and DEMs, which is not a cost effective and elegant solution. In the future, we will incorporate the whole data processing pipeline into PhotoScan so the process can be streamlined and results can be directly generated by one software.

## Conclusion

In this paper, we developed a methodology of using multispectral images acquired by an UAS to measure cotton plant height, canopy cover, and vegetation index, as well as to detect flowers, which is an important step towards bringing UAS and advanced sensor technologies to cotton breeding. The ground calibration targets were effective to substantially reduce systematic errors in the digital elevation model due to georeferencing errors caused by the low-accuracy GPS on the UAS and lack of accurately surveyed ground control points. Histograms of plot DEM presented more information about the distribution of plant canopy height than the maximum height, and can be used to derive statistics to characterize the plot height. Correlation between the canopy cover and NDVI was found, and gradually declined after the canopy fully closed. The difference in the spectral response among ground, leaves, and cotton flowers showed the potential of detecting cotton flowers using multispectral images, which can be used in the future to assess the timing of cotton flowering and its distribution over time. Although we only demonstrated the application of aerial multispectral images for cotton phenotyping in this study, we will integrate a color camera and thermal camera into the UAS in the future. The combination of color, multispectral, and thermal images could provide a wide range of information about crops, which can be used to measure more complicated traits for cotton and other crops alike.

## Supporting information

S1 Fig Ground calibration target patterns. (TIF)

S2 Fig Canopy cover (A) and NDVI (B) for plots in rows 7 to 12 on different dates. Each square is one plot. The color indicates values of canopy cover (in panel A) and NDVI (in panel B), where hot color means high value and cool color means low value.
(TIF)

S1 Table Multiple comparison tests among genotypes for each dataset’s calculated maximum height and manually measured maximum height. Green color indicates no significant statistic different between the two genotypes and red color indicates significant statistic different (p<0.05).
(DOCX)
