# Supplementary figures and images for: Multispectral imaging and unmanned aerial systems for cotton plant phenotyping
Source: PLoS One. 2019 Feb 27;14(2):e0205083. doi: 10.1371/journal.pone.0205083 (PMC6392284; doi:10.1371/journal.pone.0205083)

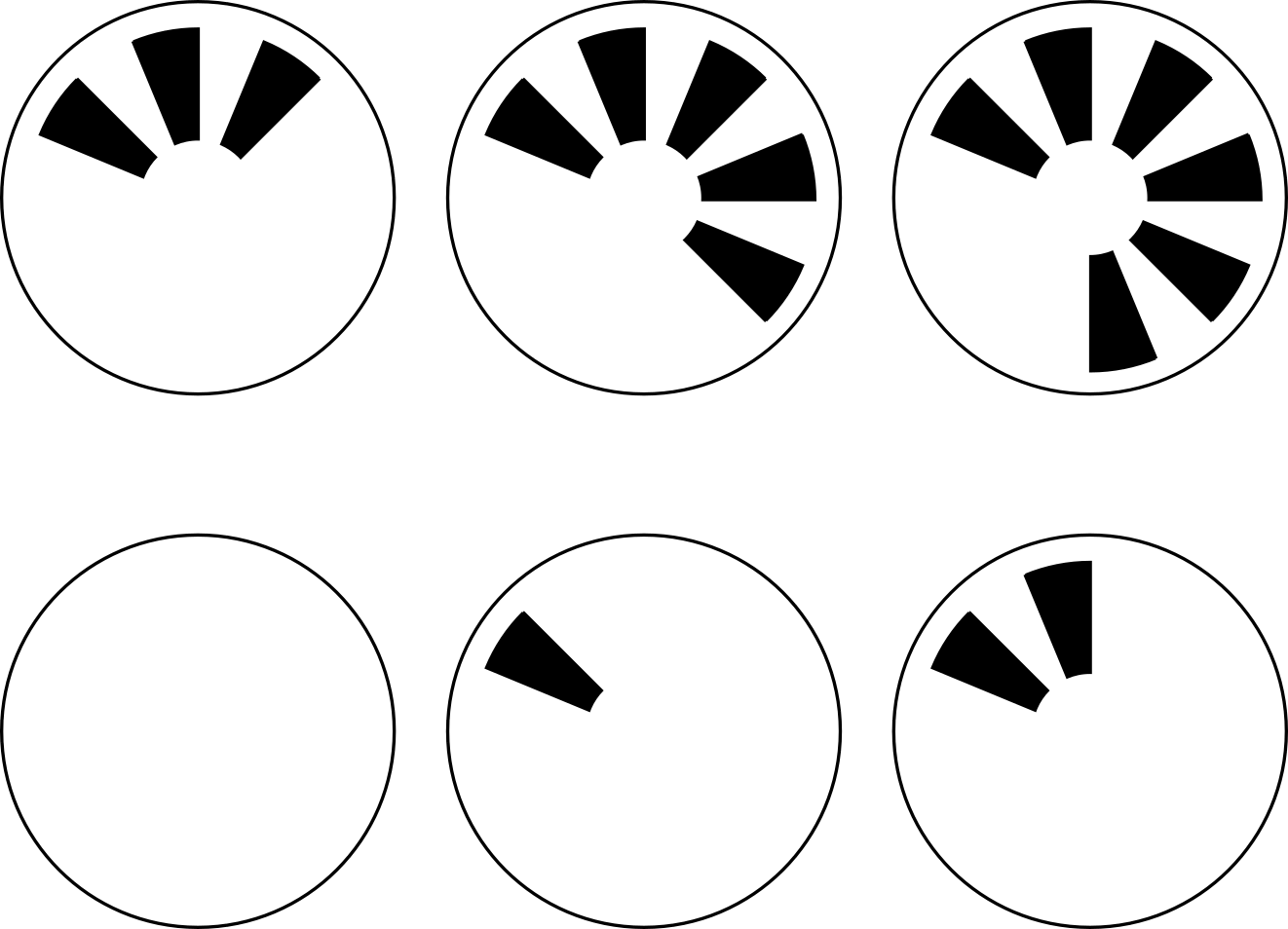

Supplement: S1 Fig — (TIF) [file pone.0205083.s001.tif]

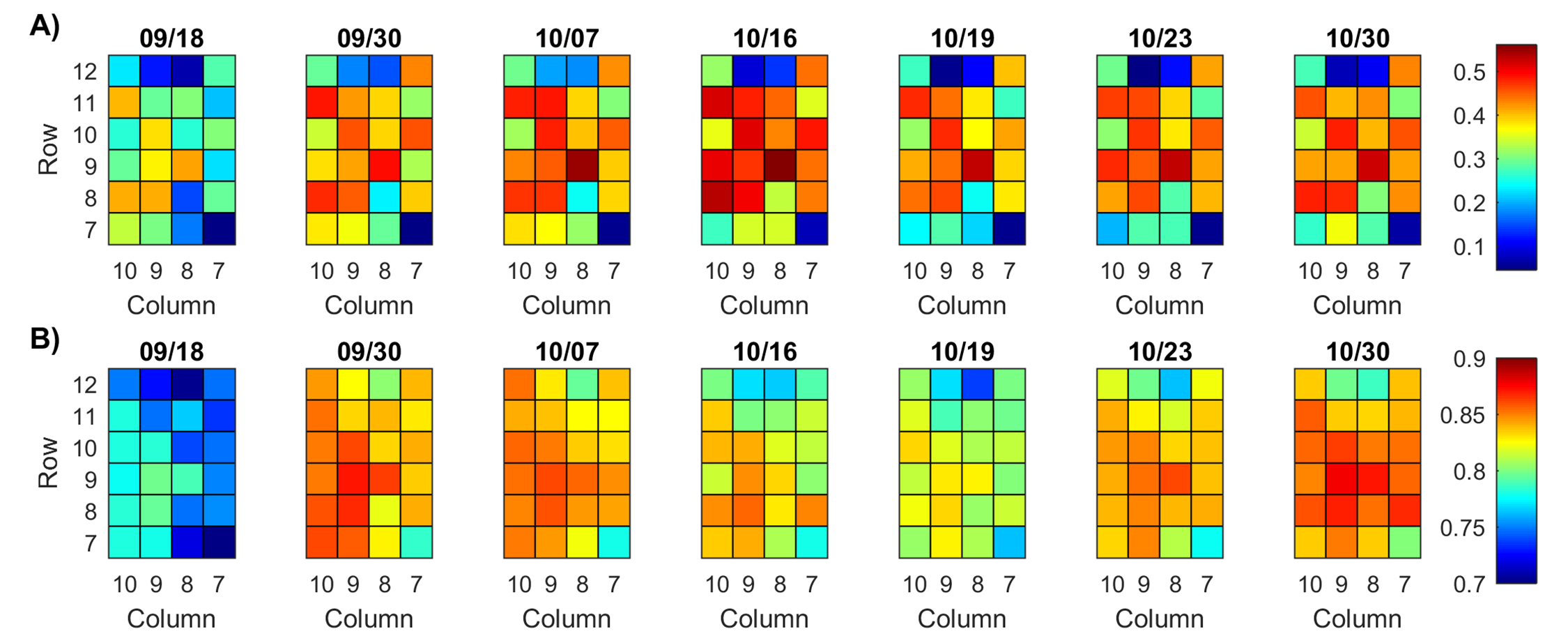

Supplement: S2 Fig — Each square is one plot. The color indicates values of canopy cover (in panel A) and NDVI (in panel B), where hot color means high value and cool color means low value. (TIF) [file pone.0205083.s002.tif]
