# Supplementary material for: Multispectral imaging and unmanned aerial systems for cotton plant phenotyping
Source: PLoS One. 2019 Feb 27;14(2):e0205083. doi: 10.1371/journal.pone.0205083 (PMC6392284; doi:10.1371/journal.pone.0205083)
Supplement: S1 Table — Green color indicates no significant statistic different between the two genotypes and red color indicates significant statistic different (p<0.05). (DOCX) [file pone.0205083.s003.docx]

We performed multiple comparisons among genotypes for the maximum height and manually measured maximum height (ANOVA statistical test). The test was performed for each dataset. Because of small data sample (8 samples per genotype for dataset 09/30 and 10/07 and 4 samples per genotype for dataset 10/16, 10/19, 10/23 and 10/30), the calculated maximum height and manually measured maximum height did not consistently have the same mean separation for all the datasets. However, it is clear that manual measurement consistently showed statistical difference between genotype 2 and 5 in all data sets except one (09/30). Our UAV based system also consistently showed the difference between the two genotypes.

**S1 Table. Multiple comparison tests among genotypes for each dataset’s calculated maximum height and manually measured maximum height.** Green color indicates no significant statistic different between the two genotypes and red color indicates significant statistic different (p<0.05).

| 09/30 Calculated maximum height | | | | | | |  | 09/30 Manually measured maximum height | | | | | | |
| --- | --- | --- | --- | --- | --- | --- | --- | --- | --- | --- | --- | --- | --- | --- |
| Genotype | 1 | 2 | 3 | 4 | 5 | 6 |  | Genotype | 1 | 2 | 3 | 4 | 5 | 6 |
| 1 |  |  |  |  |  |  |  | 1 |  |  |  |  |  |  |
| 2 |  |  |  |  |  |  |  | 2 |  |  |  |  |  |  |
| 3 |  |  |  |  |  |  |  | 3 |  |  |  |  |  |  |
| 4 |  |  |  |  |  |  |  | 4 |  |  |  |  |  |  |
| 5 |  |  |  |  |  |  |  | 5 |  |  |  |  |  |  |
| 6 |  |  |  |  |  |  |  | 6 |  |  |  |  |  |  |
| 10/07 Calculated maximum height | | | | | | |  | 10/07 Manually measured maximum height | | | | | | |
| Genotype | 1 | 2 | 3 | 4 | 5 | 6 |  | Genotype | 1 | 2 | 3 | 4 | 5 | 6 |
| 1 |  |  |  |  |  |  |  | 1 |  |  |  |  |  |  |
| 2 |  |  |  |  |  |  |  | 2 |  |  |  |  |  |  |
| 3 |  |  |  |  |  |  |  | 3 |  |  |  |  |  |  |
| 4 |  |  |  |  |  |  |  | 4 |  |  |  |  |  |  |
| 5 |  |  |  |  |  |  |  | 5 |  |  |  |  |  |  |
| 6 |  |  |  |  |  |  |  | 6 |  |  |  |  |  |  |
| 10/16 Calculated maximum height | | | | | | |  | 10/16 Manually measured maximum height | | | | | | |
| Genotype | 1 | 2 | 3 | 4 | 5 | 6 |  | Genotype | 1 | 2 | 3 | 4 | 5 | 6 |
| 1 |  |  |  |  |  |  |  | 1 |  |  |  |  |  |  |
| 2 |  |  |  |  |  |  |  | 2 |  |  |  |  |  |  |
| 3 |  |  |  |  |  |  |  | 3 |  |  |  |  |  |  |
| 4 |  |  |  |  |  |  |  | 4 |  |  |  |  |  |  |
| 5 |  |  |  |  |  |  |  | 5 |  |  |  |  |  |  |
| 6 |  |  |  |  |  |  |  | 6 |  |  |  |  |  |  |
| 10/19 Calculated maximum height | | | | | | |  | 10/19 Manually measured maximum height | | | | | | |
| Genotype | 1 | 2 | 3 | 4 | 5 | 6 |  | Genotype | 1 | 2 | 3 | 4 | 5 | 6 |
| 1 |  |  |  |  |  |  |  | 1 |  |  |  |  |  |  |
| 2 |  |  |  |  |  |  |  | 2 |  |  |  |  |  |  |
| 3 |  |  |  |  |  |  |  | 3 |  |  |  |  |  |  |
| 4 |  |  |  |  |  |  |  | 4 |  |  |  |  |  |  |
| 5 |  |  |  |  |  |  |  | 5 |  |  |  |  |  |  |
| 6 |  |  |  |  |  |  |  | 6 |  |  |  |  |  |  |
| 10/23 Calculated maximum height | | | | | | |  | 10/23 Manually measured maximum height | | | | | | |
| Genotype | 1 | 2 | 3 | 4 | 5 | 6 |  | Genotype | 1 | 2 | 3 | 4 | 5 | 6 |
| 1 |  |  |  |  |  |  |  | 1 |  |  |  |  |  |  |
| 2 |  |  |  |  |  |  |  | 2 |  |  |  |  |  |  |
| 3 |  |  |  |  |  |  |  | 3 |  |  |  |  |  |  |
| 4 |  |  |  |  |  |  |  | 4 |  |  |  |  |  |  |
| 5 |  |  |  |  |  |  |  | 5 |  |  |  |  |  |  |
| 6 |  |  |  |  |  |  |  | 6 |  |  |  |  |  |  |
| 10/30 Calculated maximum height | | | | | | |  | 10/30 Manually measured maximum height | | | | | | |
| Genotype | 1 | 2 | 3 | 4 | 5 | 6 |  | Genotype | 1 | 2 | 3 | 4 | 5 | 6 |
| 1 |  |  |  |  |  |  |  | 1 |  |  |  |  |  |  |
| 2 |  |  |  |  |  |  |  | 2 |  |  |  |  |  |  |
| 3 |  |  |  |  |  |  |  | 3 |  |  |  |  |  |  |
| 4 |  |  |  |  |  |  |  | 4 |  |  |  |  |  |  |
| 5 |  |  |  |  |  |  |  | 5 |  |  |  |  |  |  |
| 6 |  |  |  |  |  |  |  | 6 |  |  |  |  |  |  |
